# Unraveling Health Risk and Speciation of Arsenic from Groundwater in Rural Areas of Punjab, Pakistan

**DOI:** 10.3390/ijerph121012371

**Published:** 2015-10-05

**Authors:** Muhammad Bilal Shakoor, Nabeel Khan Niazi, Irshad Bibi, Mohammad Mahmudur Rahman, Ravi Naidu, Zhaomin Dong, Muhammad Shahid, Muhammad Arshad

**Affiliations:** 1 Institute of Soil and Environmental Sciences, University of Agriculture Faisalabad, Faisalabad 38040, Pakistan; E-Mails: bilalshakoor88@gmail.com (M.B.S.); irshad.niazi81@gmail.com (I.B.); arshad_ises@yahoo.com (M.A.); 2 Southern Cross GeoScience, Southern Cross University, Lismore 2480, NSW, Australia; 3 Centre for Environmental Risk Assessment and Remediation (CERAR), Mawson Lakes Campus, University of South Australia, SA 5095, Australia; E-Mail: mohammad.rahman@unisa.edu.au; 4 Global Centre for Environmental Remediation (GCER), Faculty of Science and Information Technology, The University of Newcastle, University Drive, Callaghan, NSW 2308, Australia; E-Mails: ravi.naidu@newcastle.edu.au (R.N.); morrow.dong@newcastle.edu.au (Z.D.); 5 Cooperative Research Centre for Contamination Assessment and Remediation of the Environment (CRC CARE), P.O. Box 486, Salisbury South, SA 5106, Australia; 6 Department of Environmental Sciences, COMSATS Institute of Information Technology, Vehari 61100, Pakistan; E-Mail: muhammadshahid@ciitvehari.edu.pk

**Keywords:** arsenic, groundwater, speciation, health risk, cancer, contamination, toxicity

## Abstract

This study determined the total and speciated arsenic (As) concentrations and other health-related water quality parameters for unraveling the health risk of As from drinking water to humans. Groundwater samples (*n* = 62) were collected from three previously unexplored rural areas (Chichawatni, Vehari, Rahim Yar Khan) of Punjab in Pakistan. The mean and median As concentrations in groundwater were 37.9 and 12.7 µg·L^−1^ (range = 1.5–201 µg·L^−1^). Fifty three percent groundwater samples showed higher As value than WHO safe limit of 10 µg·L^−1^. Speciation of As in groundwater samples (*n* = 13) showed the presence of inorganic As only; arsenite (As(III)) constituted 13%–67% of total As and arsenate (As(V)) ranged from 33% to 100%. For As health risk assessment, the hazard quotient and cancer risk values were 11–18 and 46–600 times higher than the recommended values of US-EPA (*i.e*., 1.00 and 10^−6^, respectively). In addition to As, various water quality parameters (e.g., electrical conductivity, Na, Ca, Cl^−^, NO_3_^−^, SO_4_^2−^, Fe, Mn, Pb) also enhanced the health risk. The results show that consumption of As-contaminated groundwater poses an emerging health threat to the communities in the study area, and hence needs urgent remedial and management measures.

## 1. Introduction

Contamination of groundwater aquifers with arsenic (As) is a global health issue because As is a toxic element and has been classified as a Group A human carcinogen [1]. Arsenic is a metalloid and a member of Group VA of the periodic table. It has an atomic number of 33, atomic weight of 74.9216 g·mol^−1^ and an electronic configuration of [Ar]^18^ 4s^2^ 3d^10^ 4p^3^ [2]. In aquatic environments, As is mainly found in arsenite (As(III), as AsO_3_^3−^) and arsenate (As(V), as AsO_4_^3−^) oxyanionic forms; the redox potential and pH play an important role in determining the chemical forms of As. At high pH and under reducing conditions, As(III) is the predominant species, while As(V) is the major species in an oxidizing environment, at low pH [3]. The dissociation constant (pK) values for As(V) range from 2.20 to 11.53, while for As(III) pK values range between 9.22 and 13.40. The toxicity of As depends on its chemical species. Inorganic forms of As (As(III), As(V)) are more toxic than the organo-As forms (e.g., monomethyl arsenate (MMAA)) [4].

Arsenic is released into the aquatic environments through the natural processes (such as weathering reactions, volcanic emissions) and anthropogenic activities for instance, petroleum refining, use of wood preservatives, leather tanning operation, agricultural use of pesticides and herbicides [5,6,7].

Arsenic contamination of groundwater (up to 5000 μg·L^−1^) has been reported worldwide in over 105 countries [8]. It has been estimated that over 200 million people in the world are at the health risk of As poisoning due to ingestion of As-rich water. Arsenic affected countries (with their corresponding maximum permissible limits) include the USA (10 μg·L^−1^), Canada (10 μg·L^−1^), Poland (10 μg·L^−1^), Japan (10 μg ·L^−1^), Australia (10 µg·L^−1^), Taiwan (10 µg·L^−1^), Vietnam (10 µg·L^−1^), Brazil (50 µg·L^−1^), Hungary (50 µg·L^−1^), Mexico (50 µg·L^−1^), Argentina (50 µg·L^−1^), Nepal (50 µg·L^−1^), Laos (50 µg·L^−1^), Myanmar (50 µg·L^−1^), Cambodia (50 µg·L^−1^), China (50 µg·L^−1^), Chile (50 µg·L^−1^), Bangladesh (50 µg·L^−1^), India (50 µg·L^−1^) and Pakistan (50 µg·L^−1^) (Table S1, Supplementary Material) [9,10]. Given the highly toxic nature of As and widespread As contamination, the World Health Organization (WHO) has set an As safe limit of 10 µg·L^−1^ in drinking water [11].

Bangladesh and several states in India where people rely on As-contaminated groundwater for drinking, cooking and also to irrigate food crops such as rice and vegetables have seen the worst As induced poisoning of the century [12,13]. In Bangladesh, 59 out of 64 districts that have been investigated display As levels above the WHO safe limit of 10 µg·L^−1^ [14]. The maximum As levels in South 24 Parganas (India) and Noakhali (Bangladesh) were observed to be 3700 µg·L^−1^ and 4730 µg·L^−1^, respectively [15,16]. According to an estimate, about 80 million people in Bangladesh alone and 43 million people in (West Bengal) India have been exposed to health risks from As-contaminated drinking water [17].

The people exposed to chronic As poisoning through drinking water can show dermatological symptoms such as keratosis (dry, rough, papular skin lesions) and melanosis (change pigmentation) [4,18]. Arsenic toxicity may also result in neurological, reproductive, cardiovascular, hepatic, hematological, respiratory and diabetic effects in humans. Chronic exposure to high level of As is linked to the increased risk of carcinogenicity such as lung, bladder, liver, skin and kidney cancers [4].

Arsenic health risk can be calculated from As-contaminated water and food, the two major exposure routes of As in humans [19]. From food, health risk assessment can be measured in terms of estimated daily intake (EDI) by evaluating the exposure dose of As due to the consumption of food crops. However, health risk assessment based on consumption of As-contaminated water is highly significant due to the direct, intense and continuous human exposure to As. Arsenic concentration in drinking water is used to calculate potential health risk assessment (chronic and carcinogenic effects) such as, average daily dose (ADD), hazard quotient (HQ), and carcinogenic risk (CR) [20,21,22,23]. Hazard quotient and CR are considered to be present if the values are HQ > 1.00 and CR > 10^−6^ [24].

Arsenic health risk assessment was previously estimated based on total As in water or in food crops in Pakistan. In the Khyber Pakhtunkhwah province of Pakistan, the As health risk assessment was calculated by Muhammad *et al.* [25], who reported HQ values >1 in the study area. Arain *et al.* [26] calculated the ADD of As in the food to be 9–12 µg·kg^−1^ day^−1^ in Sindh and Punjab provinces (only in urban areas of Lahore and Kasur). Sultana *et al.* [27] documented that 87% water samples in Lahore exceeded the typical HQ values of 1.00 (HQ = 2.3–48.6) in Punjab.

In Pakistan, a study in Muzaffargarh district demonstrated that the natural (oxidation of As-rich minerals) as well as anthropogenic sources (use of fertilizers) resulted in a release of As in groundwater up to 906 μg·L^−1^ [28]. A few more studies investigating groundwater As concentration have been carried out in Lahore and Kasur (As levels up to 1900 μg·L^−1^) districts of Punjab [27,29,30,31,32]; in Jamshoro (As up to 106 μg·L^−1^) [33] and Tharparkar (As up to 2580 μg·L^−1^) districts of Sindh [34,35]. To the best of our knowledge, aqueous phase speciation of As (As(III) and As(V)) has not been determined by using advanced hyphenated techniques such as ion chromatography (IC) coupled with inductively coupled plasma mass spectrometry (ICP-MS) in the previously unexplored study area of Punjab, Pakistan (as we examined in this study).

To our understanding, investigating the speciated and total As contents and assessing the human health risk from intake of As-contaminated water are the critically-important and existing gaps to resolve the emerging issue of As-contamination in Punjab (Pakistan). The primary and specific objectives of the present study were to: (i) determine the total As content and species of As in groundwater from three previously unexplored areas of Punjab (Pakistan); (ii) evaluate the concentration of various water quality parameters such as major cations, anions and trace metals, and (iii) estimate the health risk of As by calculating ADD, HQ and CR values for As-contaminated drinking water by US-EPA model and bootstrap method.

## 2. Experimental Section

### 2.1. Topography

**Figure 1:**
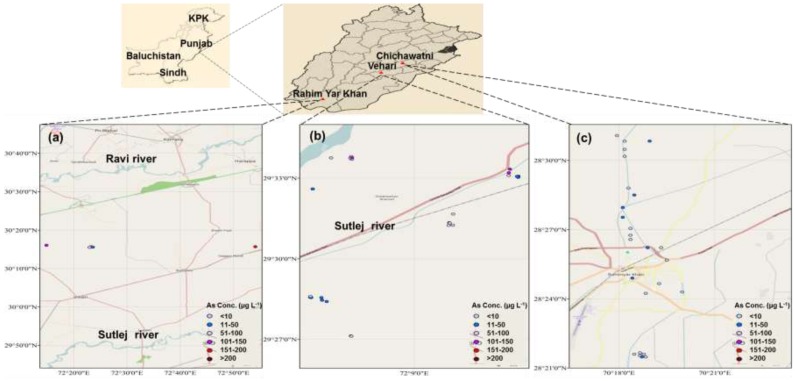
Map of the study area showing locations of groundwater sampling for As (µg·L^−1^) in Punjab, Pakistan; (**a**) Chichawatni; (**b**) Vehari; (**c**) Rahim Yar Khan.

The study was conducted in three rural areas (Chichawatni, Vehari, Rahim Yar Khan) in Punjab, Pakistan. Chichawatni is a city in Sahiwal district of Punjab, Pakistan. It is situated 30°53′33″ N and 72°70′00″ E (Figure 1a). It lies approximately 45 km away from Sahiwal and its population is approximately 120,000 [36]. This city has a semi-arid alluvium plain area with the exception of a few belts of ravines and uneven land formed by gully erosion along the lower Bari Doab and its distributaries. The River Ravi flows on its north-west and the River Sutlej (Figure 1a) on its south [36].

Vehari is situated between 30°04′19″ N and 72°35′28″ E (Figure 1b) with a population of 7,000,000. Literally, Vehari means low lying riverine settlement. It is situated on right bank of the River Sutlej in the heart of Nili Bar which is between Ravi, Bias and Sutlej Rivers (Figure 1b). Vehari consists of alluvium plain area with fertile land which is irrigated with the fertile water of Chenab and Ravi Rivers [37].

Rahim Yar Khan is situated in the alluvium plain between Indus River in the west and Cholistan desert in the east. The city lies between 28°42′00″ N and 70°30′00″ E (Figure 1c) with the population of 240,000. This city is divided into three main physical features: (i) riverine area. (ii) canal irrigated area, and (iii) desert area (Cholistan). The riverine area of Rahim Yar Khan lies close to the Indus River mainly falling in the river bed [38].

### 2.2. Groundwater Sampling

Groundwater samples (*n* = 62) were collected from hand-pumps, electric pumps and tube wells at depths of 9–107 m (Table 1) around three rural areas (Chichawatni, Vehari and Rahim Yar Khan) of Punjab in Pakistan. All pumps/wells were flushed for 5 min to obtain fresh groundwater prior to collect water sample. The groundwater samples (100 mL each) were taken in triplicate in three separate plastic bottles having airtight caps. One water sample was acidified on-site by adding 2–3 drops of concentrated nitric acid (HNO_3_) to stabilize As and metal ions and reduce their precipitation [39]. The acidified water samples were used to analyse total As, iron (Fe), manganese (Mn), nickel (Ni), lead (Pb), cadmium (Cd), copper (Cu), silicon (Si), cobalt (Co), aluminum (Al), zinc (Zn), boron (B), chromium (Cr), phosphorus (P), sulfur (S) and major cations including calcium (Ca), magnesium (Mg), potassium (K) and sodium (Na). The second water sample was kept non-acidified to analyse various anions including carbonates (CO_3_^2−^), bicarbonates (HCO_3_^−^), chloride (Cl^−^), nitrate (NO_3_^−^), sulfate (SO_4_^2−^), phosphate (PO_4_^3−^) and fluoride (F^−^). For As speciation (Table 2), the third sample was preserved with 0.025 M ethylenediaminetetraacetic acid (EDTA) [40]. Samples were kept in insulated cooler containing ice, transported to the laboratory and stored at 4 °C, then transported to Centre for Environmental Risk Assessment and Remediation (CERAR) laboratory, University of South Australia, Australia by FedEx courier with dry ice under strict quarantine regulations and stored at 4 °C prior to various chemical analyses.

### 2.3. Analytical Methods

The electrical conductivity (EC) and pH of water samples were measured *in situ* by using an EC meter (Model 470, Jenway, Stone, Staffordshire, U.K.) and a pH meter (Model 370, Jenway), respectively (Table 1). Total dissolved solids (TDS) were measured by using a TDS meter (Model 3000c, Ohaus, Parsippany, NJ, USA). An inductively coupled plasma mass spectrometer (ICP-MS; Model 7500ce, Agilent, Santa Clara, CA, USA) with an octopole reaction system (ORS) was used for multi-elements analysis (total As, Al, Cr, Ca, Mg, Na, Fe, P, K, Ni, Pb, Mn, Cd, Cu, Si, Co, Zn, B, S) (Table 3). For anions (NO_3_^−^, SO_4_^2−^, F^−^, Cl^−^, PO_4_^3−^) analyses in water samples, ion chromatography (IC, Model ICS 2000, Dionex, Carlsbad, CA, USA) was used. Arsenic speciation was done by using an IC-ICP-MS. Other anions such as CO_3_^2−^ and HCO_3_^−^ were determined by acid titration (H_2_SO_4_) using phenolphthalein, and methyl orange as indicators [41]. Calcium hardness, Mg hardness and total hardness as CaCO_3_ were calculated following the methodology described by Spellman [42].

### 2.4. Quality Control

A standard reference material (SRM) from the National Institute of Standard and Technology (NIST, Gaithersburg, MD, USA) such as SRM 1640 (trace elements in natural water) was used to check accuracy of the analytical results for As. The concentration of As in SRM 1640 was estimated by following the same analysis procedure as for the groundwater samples. The certified concentration of As in NIST SRM 1640 was 26.67 ± 0.41 μg·L^−1^. From our ICP-MS results, the mean As concentration of NIST SRM 1640 was detected as 27.32 ± 0.65 μg·L^−1^ (*n* = 8) with a recovery of 104.21%.

### 2.5. Water Consumption Patterns of People in the Study Area

In the study area, average water intake rates were 2.1 and 1.9 L per day for adult males and females, respectively (data not shown). The average daily water intake in our study was almost similar to the findings of Sultana *et al.* [27], Brahman *et al.* [43] and Kazi *et al.* [44], who reported 2 L·day^−1^ of average drinking water intake in Punjab (Lahore and Kasur) and Sindh (Nagarparkar and Manchar lake) provinces of Pakistan. However, in the current study the average daily water intake rate was less than the results of Rahman *et al.* [45] in Bangladesh (3.2 and 2.7 L·day^−1^ for adult males and females, respectively) and Mondal *et al.* [46] in West Bengal, India (3.1 and 2.6 L·day^−1^ for adult males and females, respectively).

### 2.6. Human Health Risk Assessment

#### 2.6.1. Exposure Assessment

The human health risk assessment model derived by US-EPA Equations (1–3) was used to evaluate the toxic effects of As present in drinking water on the health of people in three study locations [47]. The health risk assessment was done to estimate the probability of individuals being exposed to As poisoning from drinking water. For this purpose, average daily dose (ADD) of As due to the intake of As-contaminated drinking water was calculated by the Equation (1):

(1) $$ADD=\frac{C\times IR\times ED\times EF}{BW\times AT}$$

where:

C = Concentration of As in water (mg·L^−1^)
IR = Water ingestion rate (L·day^−1^)
ED = Exposure duration (assumed 67 years to make a comparison with previous studies from Pakistan and other countries)
EF = Exposure frequency (365 days year^−1^)
BW = Body weight (72 kg) [27]
AT= Average life time (24,455 days)

#### 2.6.2. Human Health Risk Assessment

Chronic and carcinogenic risk levels were also determined in individuals of the study area. The hazard quotient (HQ) was computed by the Equation (2) [47]:

(2) $$HQ=\frac{ADD}{RfD}$$

where RfD represents oral reference dose (0.0003 mg·kg^−1^·day^−1^) for As calculated by US-EPA [24]. Cancer risk (CR) was calculated by using the Equation (3):

(3) $$CR=\frac{ADD}{CSF}$$

where CSF is the cancer slope factor for As which is 1.5 mg·kg^−1^·day^−1^, according to US-EPA [24].

### 2.7. Carcinogenic and Non-Carcinogenic Risk

Considering the potential cancer risk from As-contaminated groundwater (used as drinking water in the study area, the cancer probability for As is also calculated as described in our earlier study for Bangladesh and India [23] Equation (4):

(4) $$R_{i}=1/5000\times \sum_{j=1}^{5000}C_{ij}\times k_{j}$$

where the subscript *i* represents the sampling regions, *C×j* is the average concentration of As in groundwater that was estimated by the bootstrap method, *j* is the sampling time in the bootstrap method, and *k_j_* is the random number of sampling from the distribution for cancer slope of As for drinking water. The cancer slope (5 × 10^−5^ per μg·L^−1^, with the range of 3 × 10^−5^ per μg·L^−1^ to 7 × 10^−5^ per μg·L^−1^) recommended by US-EPA was used in this study [48]. In this study, the non-carcinogenic risk was also estimated by using the bootstrap (re-sampling) method as described for cancer risk. The value of 3 × 10^−4^ mg·kg^−1^ was recommended as reference dose.

### 2.8. Statistical Analysis

Basic descriptive statistical analyses were done by using JMP version 9.0 software and the maps were prepared using the Arc GIS 9.2 computer package [45].

## 3. Results and Discussion

### 3.1. Total and Speciated As Contents

Figure 1 shows the distribution of As concentrations in groundwater and locations of the study area. Arsenic concentration ranged 1.5–201 µg·L^−1^ (Table 1; Figure S1, Supplementary Material) in groundwater samples of the study area. Sixty two percent groundwater samples showed As levels 15–19 times (Table S2, Supplementary Material) higher than the safe limit (10 µg·L^−1^) set by the WHO, US-EPA and Health Canada (HC) (Table S3, Supplementary Material). In Chichawatni (CW), 100% samples, in Vehari (Vh) 66% and in Rahim Yar Khan (RYK) 20% samples contained 2–19 times higher As concentrations as compared with the safe limits set by the WHO, US-EPA and HC (Table S3, Supplementary Material). Groundwater samples of CW (88%) and Vh (60%) showed 2–4 times greater As concentration than Pakistan-Environmental Protection Agency (Pak-EPA) permissible limit for As (50 µg·L^−1^).

Arsenic speciation data of the selected groundwater (*n* = 13) samples (*i.e*., having maximum total As concentration) in CW, Vh and RYK showed that As(III) constituted 13% to 67% of total As and As(V) ranged from 33% to 100% (Table 2). High proportion of As(V) in groundwater samples indicated that the subsurface sediments and aquifers would have been oxidized over time due to continuous pumping of groundwater [30]. Also, presence of high HCO_3_^−^ (>250 mg·L^−1^) and SO_4_^2−^ (>1400 mg·L^−1^) concentrations supports oxidation of sediments and aquifers [3]. Enrichment of groundwater with As may arise by oxidation of As containing minerals such as arsenopyrite (FeAsS) thus discharging soluble As, SO_4_^2−^ and ferric iron (Fe(III)) or by the reduction of iron oxides resulting in the release of ferrous iron (Fe(II)), As(III) and As(V) in the groundwater [49,50]. Further research is warranted to determine the exact mechanism of As release in the groundwater of the study area.

The maximum As concentration in groundwater was higher than the maximum As concentration (96 μg·L^−1^) reported by Arain *et al.* [26] and Baig *et al.* [33] (106 μg·L^−1^) in Jamshoro (Sindh) groundwater. Nevertheless, this study first time assessed the nature of As species present in the groundwater of Punjab, which could be used to better characterize the risks posed by consuming As-contaminated water in the previously unexplored study area.

**Figure 2:**
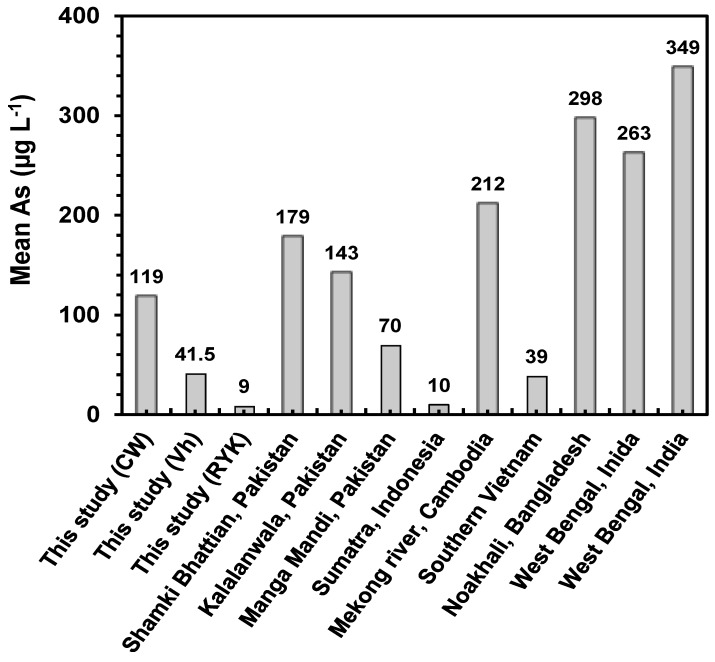
Comparative mean As concentrations (µg·L^−1^) in groundwater recorded in this study with other studies conducted in Pakistan (Sultana *et al.* [24]), Indonesia (Wnikle *et al.* [46]), Cambodia (Buschmann *et al.* [47]), Vietnam (Buschmann *et al.* [47]), Bangladesh and India (Rahman *et al.* [18]).

Figure 2 demonstrates the mean As concentrations found in this study and the earlier studies in order to compare the extent of groundwater As contamination. Mean As concentrations found in our study were higher than the As concentrations reported previously in Pakistan [30], Indonesia [51] and Vietnam [52], but less than the concentrations documented in Cambodia, Bangladesh and India [23,52].

**Table 1:** Summary statistics of anions and hydrochemical water quality parameters in groundwater samples collected from three rural areas of Punjab, Pakistan.

Parameter	Chichawatni (CW, *n* = 8)				Vehari (Vh, *n* = 28)				Rahim Yar Khan (RYK, *n =* 26)			
	Mean	Median	Range	S.D (±)	Mean	Median	Range	S.D (±)	Mean	Median	Range	S.D (±)
As (µg·L^−1^)	95	90	23–201	60.52	41.5	22.12	1.5–144	45.61	9.2	6	1.66–31	8
pH	6.84	7.4	2.83–7.98	1.65	7.36	7.52	3.01–8.06	0.87	7.6	7.7	6.9–8.35	0.4
EC (dS·cm^−1^)	0.17	0.16	0.14–0.3	0.05	0.1	0.08	0.03–0.5	0.08	0.08	0.06	0.02–0.34	0.07
Ca hardness	168	157	91–277	61	152	145	73–230	45.6	152	90	35–569	130
Mg hardness	148	152	80–188	39	115.5	105	12–260	54	138	109	36–339	93.5
Total hardness	316	330	171–461	91	268	254	85–474	90	291	207	72–908	213
TDS	1.77	1.65	1.44–2.82	0.46	1	0.8	0.3–5	0.83	0.8	0.7	0.23–3.35	0.7
SAR	5.6	7	0.006–9	2.8	2.5	2.2	0.35–6.4	1.3	4.6	2.9	0.7–16	4
CO_3_^2−^ (mg·L^−1^)	26.4	26.4	19–36	5.9	25.5	19.2	7.2–108	23.5	33.6	28.8	7.2–85.2	20.2
HCO_3_^−^ (mg·L^−1^)	104.62	75.6	52.5–215	63.7	95	72.59	33–277	63	56.2	50.6	9.8–133	26.8
NO_3_^−^ (mg·L^−1^)	1189	1110.6	632.5–2229	490	1395	1457	0–2365	581	2250.1	2275.3	0–5370	1309.9
SO_4_^2−^ (mg·L^−1^)	1002	935.5	751–1447	240.7	277	223.95	0–752	170	261.3	180.2	20.4–1082	252.9
Cl^−^ (mg·L^−1^)	194	183.5	69–381	100	92	88.75	7–218	50.5	244.2	156.6	39–902	220.2
F (mg·L^−1^)	0	0	0	0	0.004	0	0–0.09	0.02	0.002	0	0–0.045	0.009

As, Arsenic; EC, electrical conductivity; Ca, Calcium; Mg, Magnesium; TDS, Total dissolved solids; SAR, Sodium Adsorption Ratio; CO_3_^2−^, Carbonates; HCO_3_^−^, Bicarbonates; NO_3_^−^, Nitrate; SO_4_^2−^, Sulfate; Cl^−^, Chloride; F, Fluoride; S.D (±), Standard Deviation.

**Table 2:** Speciated As contents in selected groundwater samples (*n* = 13) collected from three rural areas of Punjab, Pakistan.

Sample Name	As(III) (%)	As(V) (%)
CW1	29	71
CW2	40	60
CW3	33	67
CW4	48	52
CW5	46	54
CW8	55	45
Vh21	0	100
Vh22	43	57
Vh28	0	100
Vh34	17	83
RYK42	67	33
RYK55	34	66
RYK56	13	87

CW, Chichawatni; Vh, Vehari; RYK, Rahim Yar Khan; As(III), arsenite; As(V), arsenate.

### 3.2. Groundwater Composition/Chemistry

The pH and EC values of groundwater samples are shown in Table S2, Supplementary Material. The mean values of pH were 7.4, 7.52 and 7.60 in groundwater samples of CW, Vh and RYK, respectively (Table 1). The pH of all the groundwater samples was within the range of permissible limit (6.5–8.5) set by WHO (Supplementary Table S3, Supplementary Material). The data showed that groundwater was slightly alkaline and the dissolved carbonates were predominantly in the form of bicarbonates (HCO_3_^−^) [53].

In all groundwater samples, EC values ranged from 0.14 to 0.5 dS·cm^−1^ (Table 1) in the study area, which were 125-fold higher than the WHO permissible limit (0.004 dS·cm^−1^). The maximum EC value (0.5 dS·cm^−1^) was found in the water sample from a hand pump in Vh. The excessive level of soluble salts in groundwater of the area could possibly be due to the dissolution of carbonate minerals such as aragonite [CaCO_3_], calcite [CaCO_3_], dolomite [CaMg(CO_3_)_2_] and siderite (FeCO_3_) from indigenous soil and bed rock [54]. 

Calcium hardness (35–569 mg·L^−1^), Mg hardness (12–339 mg·L^−1^) and total hardness (72–908 mg·L^−1^) as CaCO_3_ are shown in Table 1. The total hardness values of groundwater samples indicated that groundwater of the study area is very hard water having concentrations >180 mg·L^−1^ (*i.e*., indication value of very hard water) [42] in most of the groundwater samples. Total dissolved solids (TDS) ranged from 0.23 to 3.35 mg·L^−1^ (Table 1) and found within the safe limit of WHO (1000 mg·L^−1^) [23]. Sodium adsorption ratio (SAR) values of groundwater samples ranged from 0.006 to 16 (Table 1). In most of the groundwater samples of study area, SAR values were greater than its permissible limit [55].

Groundwater concentrations of major cations in the study area are presented in Table 3. The results showed that in the study area, 42%, 4%, 11% and 38% groundwater samples contained higher sodium (Na), calcium (Ca), magnesium (Mg) and potassium (K) levels, respectively, compared to Pak-EPA, WHO and HC safe limits for these elements (Table S3; Supplementary Material). The data regarding major anions is presented in Table 1. Chloride (Cl^−^), nitrate (NO_3_^−^) and sulfate (SO_4_^2−^) concentrations exceeded the Pak-EPA, WHO, US-EPA and HC safe limit (Supplementary Table S3, Supplementary Material) in 20%, 63% and 99% of groundwater samples, respectively, in the study area.

### 3.3. Trace Elements

Iron was found to be less than the safe limit (0.3 mg·L^−1^) set by WHO, US-EPA and HC in most of the groundwater samples of the study area (Table 3; Table S2, Supplementary Material). However, 1 sample in CW, 3 samples in Vh and 5 samples in RYK showed higher concentrations of Fe than WHO, US-EPA and HC permissible limits (Supplementary Material Table S3,). Low Fe concentration in the groundwater samples might be due to the high positive redox potential which indicates the oxidizing conditions thus prohibits Fe dissolution as reported by Farooqi *et al.* [30] and Rasool *et al.* [56] in different areas of east (Lahore) and south (Mailsi) Punjab, Pakistan. Furthermore, presence of high SO_4_^2−^ concentration in the groundwater of the study area also supports our earlier argument (oxidized environment).

Lead (Pb) concentration was also higher than the WHO safe limit (Table S4, Supplementary Material) in the groundwater samples of CW (67%), Vh (67%) and RYK (81%) (Table 3). Groundwater Pb contamination might be the result of leaching from plumbing system and transportation [57]. After ingestion, Pb tends to accumulate in the skeleton, brain, kidneys leading to subencephalopathic, neurological and behavioral disorders [58,59]. Manganese (Mn) concentration ranged from 0.004 to 0.8 mg·L^−1^, however, only six groundwater samples exceeded the previous WHO safe limit (0.4 mg·L^−1^) [23] and three exceeded the current Pak-EPA safe limit (0.5 mg·L^−1^) [60] of Mn in the study area. High levels of Mn can cause intelligence quotient (IQ) and nervous system damage in children. In addition, concentrations of F, Cd, Cu, Cr, Ni, Al, B and Zn (Table 1 and Table 2) were found within the safe limit set by Pak-EPA, WHO, US-EPA and HC (Table S3, Supplementary Material) in the groundwater samples of CW, Vh and RYK.

The correlation results of major cations showed significant and positive relationship for Fe–Al (*r* = 0.90, *p* < 0.05), Ca–Mg (*r* = 0.80, *p* < 0.05), Ca–Na (*r* = 0.70, *p* < 0.05) and Na–Mg (*r* = 0.72, *p* < 0.05). This may indicate that these correlated major cations have a common origin of release in the groundwater systems which could be due to geogenic sources from dissolution of their minerals [49].

### 3.4. Health Risk Assessment of As Via Drinking Water

#### 3.4.1. Potential Health Risk Assessment

Residents were interviewed about age, livelihood, literacy rate, health status and drinking water in the study area (data not shown). It was noted that most of the individuals had insufficient income, low literacy rate and lacked access to basic living facilities resulting in poor health conditions. People of the study area cannot afford safe and healthy bottled water, hence, they use hand-pump, electric pump and tube well water as the most common sources of drinking and cooking water. Therefore, these available sources of drinking water in the study area were analyzed for the potential health risk assessment through exposure assessment and risk assessment.

#### 3.4.2. Exposure Assessment and Cancer Risk Assessment

Table 4 demonstrates the health risk assessment of As in terms of ADD, HQ, and CR for the people who were exposed to the intake of As-rich groundwater. In the study area, ADD ranged from 3.6 × 10^−5^ to 5.6 × 10^−3^ mg·kg^−1^·day^−1^. The results indicate that ADD values were comparable to those calculated in Bangladesh (5.00 × 10^−2^–5.00 × 10^−1^ mg·kg^−1^·day^−1^), Turkey (2.3 × 10^−5^–5.21 × 10^−3^ mg·kg^−1^·day^−1^) and Vietnam (1.1 × 10^−3^–4.3 × 10^−3^ mg·kg^−1^·day^−1^) drinking water [20,22,61]. The ADD values in this study was greater than others reported by Muhammad *et al.* [25] (0–5.56 × 10^−7^ mg·kg^−1^·day^−1^) and Farooqi *et al.* [29] (1.1 × 10^−4^–3.7 × 10^−3^ mg·kg^−1^·day^−1^) in Khyber Pakhtunkhwah and Punjab (Pakistan). In this study As speciation (As(III/V)) data confirms that groundwater is highly contaminated with inorganic As and people are exposed to inorganic As species through intake of drinking water.

The results (Table 4) elaborate that HQ ranged from 0.12 to 18.53 with 75% samples in CW, 61% samples in Vh and 27% samples in RYK exceeded the typical toxic risk index 1.00 [27]. The maximum toxic risk index (HQ = 18.54) was noted in CW. The potential CR values ranged from 5.4 × 10^−5^ to 8.3 × 10^−3^ (Table 4) in the area under investigation. In CW the potential CR values ranged from 9 × 10^−4^ to 8 × 10^−3^, in Vh 5.54 × 10^−5^–6 × 10^−3^ and in RYK it was 6.9 × 10^−5^–2 × 10^−3^. 

**Table 3:** Summary statistics of major cations and trace metals in groundwater samples collected from three rural areas of Punjab, Pakistan.

Parameter	Chichawatni (CW, *n =* 8)				Vehari (Vh, *n =* 28)				Rahim Yar Khan (RYK, *n =* 26)			
	Mean	Median	Range	S.D (±)	Mean	Median	Range	S.D (±)	Mean	Median	Range	S.D (±)
Fe (mg·L^−1^)	0.19	0.14	0.11–0.6	0.15	0.27	0.13	0.1–2	0.42	0.2	0.2	0.01–0.6	0.1
Cr (mg·L^−1^)	0.001	0.0009	0.0003–0.002	0.0004	0.0007	0.0006	0–0.001	0.0003	0.0008	0.001	0.0003–0.002	0.0003
Si (mg·L^−1^)	8.21	8.3	6.4–10	1.1	8.5	8.64	5–10.3	1.2	9	8.8	3.9–17.5	2.8
P (mg·L^−1^)	0.01	0.007	0–0.04	0.02	0.0007	0	0–0.007	0.001	0.006	0	0–0.07	0.01
B (mg·L^−1^)	0.5	0.55	0.13–0.7	0.2	0.2	0.17	0.04–0.5	0.1	0.3	0.2	0.06–1.4	0.3
Al (mg·L^−1^)	0.004	0.001	0.00008–0.02	0.009	0.004	0.0006	0–0.03	0.001	0.0002	0.0001	0–0.001	0.0003
Ca (mg·L^−1^)	67	62.8	36–111	24.4	61	58	29.3–92	18.3	61.1	35.8	14.1–228	51.9
Mg (mg·L^−1^)	36	37	19.5–46	9.5	28	25.5	3–63	13	33.5	26.5	9–82	22.7
Na (mg·L^−1^)	308.5	364	0.3–398	132.5	117.7	110	10.2–271.5	58	246.9	125.9	27–1242	305.8
K (mg·L^−1^)	16.6	14.37	10.5–36	8	16	9	1.51–85.2	20.4	12.8	9.2	3.6–31	8.3
Pb (mg·L^−1^)	0.003	0.002	0.0008–0.006	0.002	0.003	0.002	0.0005–0.01	0.003	0.004	0.002	0.0008–0.02	0.004
Cd (mg·L^−1^)	0.0002	0.0001	0.00002–0.0004	0.0001	0.00005	0.00003	0–0.0002	0.00006	0.0001	0.00004	0–0.0006	0.0001
Cu (mg·L^−1^)	0.01	0.02	0.004–0.04	0.01	0.02	0.009	0.001–0.2	0.04	0.03	0.02	0.002–0.2	0.04
Co (mg·L^−1^)	0.0005	0.0005	0.0002–0.0008	0.0001	0.0006	0.0004	0.0001–0.002	0.0006	0.0004	0.0003	0–0.002	0.0003
Ni (mg·L^−1^)	0.006	0.005	0.002–0.01	0.002	0.005	0.004	0.001–0.009	0.001	0.006	0.006	0.002–0.02	0.004
Zn (mg·L^−1^)	0.2	0.11	0.02–0.4	0.12	0.15	0.05	0.01–2	0.4	0.3	0.2	0.05–1.5	0.3
Mn (mg·L^−1^)	0.22	0.2	0.05–0.5	0.12	0.24	0.24	0.02–0.8	0.2	0.1	0.1	0.004–0.25	0.1
S (mg·L^−1^)	176.5	177.5	106–226	39	53.55	49	6.3–128.3	31	137.4	59	13.7–827	192.1

Fe, Iron; Cr, Chromium; Si, Silicon; P, Phosphorus; B, Boron; Al, Aluminum; Ca, Calcium; Mg, Magnesium; Na, Sodium; K, Potassium; Pb, Lead; Cd, Cadmium; Cu, Copper; Co, Cobalt; Ni, Nickel; Zn, Zinc; Mn, Manganese; S, Sulfur; S.D (±), Standard Deviation.

**Table 4:** Summary of average daily dose, hazard quotient and carcinogenic risk of As in groundwater samples collected from three rural areas of Punjab, Pakistan.

Parameter		Chichawati (CW, *n* = 8)	Vehari (Vh, *n* = 28)	Rahim Yar Khan (RYK, *n* = 26)
As (µg·L^−1^)	Mean	119	41.5	9.2
	Median	118	22.12	6
	Range	23–201	1.33–144	1.66–31
	SD (±)	60.52	45.61	8
Average daily dose (mg^−1^·kg^−1^·day^−1^)	Mean	3.3 × 10^−3^	1.1 × 10^−2^	2.5 × 10^−4^
	Median	3 × 10^−3^	6.2 × 10^−4^	1.6 × 10^−4^
	Range	6 × 10^−4^–5.6 × 10^−3^	3.69 × 10^−5^–4 × 10^−3^	4.62 × 10^−5^–8 × 10^−4^
	± S.D	0.0017	0.0013	0.0003
Hazard quotient	Mean	11.01	3.84	0.85
	Median	10.88	2.05	0.55
	Range	2.16–18.58	0.13–13.36	0.15–2.87
	± S.D	5.61	4.23	0.74
Carcinogenic risk	Mean	0.005	0.0017	0.0004
	Median	0.005	0.0009	0.0003
	Range	9 × 10^−4^–8.3 × 10^−3^	5.54 × 10^−5^–6 × 10^−3^	6.9 × 10^−5^–2 × 10^−3^
	± S.D	0.0025	0.002	0.0003

*n*, No. of samples; As, Arsenic; S.D, Standard Deviation.

The CR values were higher than the US-EPA limit (10^−6^) suggesting that the people in the study area were at high carcinogenic risk. Therefore, constant monitoring of As level in groundwater is needed in order to assess if any potential health risk from consumption of As-rich water exists, thereby protecting individuals from drinking of As-contaminated water that might be harmful to their health.

### 3.5. Carcinogenic and Non-Carcinogenic Risks

In this study, a normal distribution was assumed for the cancer slope, and 95% confidence interval (CI) was estimated as the same range provided by US-EPA. Thus, the mean and standard deviation were estimated to be 5 × 10^−5^ per µg·L^−1^ and 1.03 × 10^−5^ per µg·L^−1^, respectively. Considering the variability for the exposure population, the estimated cancer risks for CW, Vh and RYK were 0.6% (95% CI: 0.4%–0.8%), 0.21% (95% CI: 0.13%–0.29%), 0.046% (95% CI: 0.032%–0.062%), respectively. The mean cancer risk was 46–600 times higher than a cancer risk of 10^−6^, which is the default acceptable level. When plus the uncertainties, the 95% CI for the three regions were estimated to be 0.31%–0.94%, 0.10%–0.34%, 0.025%–0.072%. The estimated risk presented here indicated a very high cancer risk due to the As exposure from groundwater in this region. The standard deviation’s ratio between variability and uncertainty was estimated to be 0.80%–0.92%. The HQs were estimated to be 11.36 (95% CI: 7.54–15.14), 3.95 (95% CI: 2.46–5.60) and 0.87 (95% CI: 0.61–1.18) for CW, Vh and RYK, respectively (water consumption of 2 L and body weight of 70 kg were assumed for adult). The HQs in CW and Vh were higher than 1.00, which mean that all the exposures were higher than the reference dose value [48].

Both the US-EPA model and bootstrap method demonstrated that carcinogenic and non-carcinogenic risks were found to be 46–600 and 11–18 times higher than the safe limits of 10^−6^ and 1.00, respectively, in the study area. But bootstrap method showed higher cancer risk values than the US-EPA model.

## 4. Conclusions

The results showed that 62% groundwater samples collected from various sites of CW, Vh and RYK were unfit for drinking purpose. High values of EC, Cl^−^, NO_3_^−^, SO_4_^2−^, Fe, Mn and Pb were observed in many water samples and thus do not fall under the permissible limits for these parameters. Nevertheless, parameters like CO_3_^2−^, F, Al, B, Cd, Cu, Cr, Ni and Zn were within the safe limit in all samples of study area. High concentration of As was detected in groundwater of study area. Both the US-EPA model and bootstrap method provided high carcinogenic and non-carcinogenic As risks to the individuals of the study area. The ADD and HQ were in the order of CW > Vh > RYK with the maximum HQ value of 18.54 reported in CW. The CR values were significantly (46–600 times) higher than its default acceptable level (10^−6^), thus exposing people to chronic As carcinogenicity. Higher cancer risk values were obtained by bootstrap method compared to US-EPA model. Our results demonstrated that (53%) groundwater samples contained toxic inorganic species of As(III/V) which are posing serious health risk to the people due to the intake of As-rich groundwater—the only source of drinking water in the study area. Given the health risk associated with extent of groundwater As contamination, government of Pakistan should reduce current (50 µg·L^−1^) safe limit of As to 10 µg·L^−1^, so that people of Punjab should be protected from mass poisoning of As. Additionally, in the study area future research should be focused on assessing the health risk of As in food and fodder crops that human and animals consume for their daily diet and how As can be transferred from water to crops.

## Supplementary Files

Supplementary File 1
