# Supplementary material for: Unraveling Health Risk and Speciation of Arsenic from Groundwater in Rural Areas of Punjab, Pakistan
Source: Int J Environ Res Public Health. 2015 Oct 5;12(10):12371–90. doi: 10.3390/ijerph121012371 (PMC4626974; doi:10.3390/ijerph121012371)
Supplement: Supplementary File 1 [file ijerph-12-12371-s001.pdf]

Supplementary Information

Unraveling Health Risk and Speciation of Arsenic from Groundwater in Rural Areas of Punjab, Pakistan

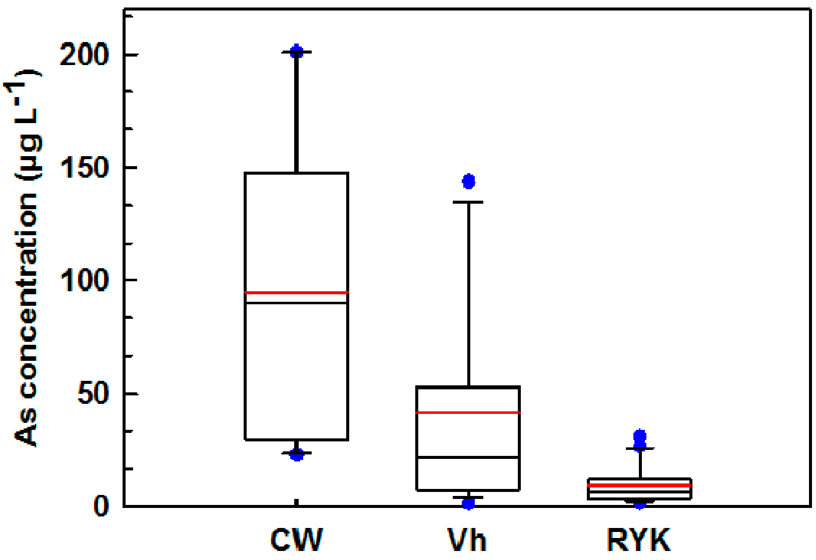

Figure S1. Arsenic concentrations in groundwater samples collected from rural areas of Punjab, Pakistan. On each box, the red mark is the mean, the black mark is median, the edges of the box are the 25th and 75th percentiles, the whiskers extend to the most extreme data points not considered outliers, and outliers are not plotted.

Table S1. Arsenic contamination level in groundwater around the world adopted from Rahman *et al.* [1] and Naidu *et al.* [2].

Country	Level of As (μg L^{-1})	National As Standard (μg L^{-1})
Pakistan	ND–906	50
Bangladesh	ND–4730	50
India	ND–3380	50
Cambodia	1–1610	50
China	50–4444	50
Myanmar	-	50
Nepal	ND–2620	50
Taiwan	0.15–3590	10
Vietnam	1–3050	10
USA	50–1700	10
Canada	>3000	10
Hungary	60–4000	50
Mexico	8–624	50
Brazil	-	50
Poland	-	10
Australia	1–5000	10
Japan	ND–293	10

As, Arsenic; ND, Not Detected.

Table S2. Concentration of As and other drinking water quality parameters in groundwater samples collected from three rural areas of Punjab, Pakistan.

Sample Name	Sample Source	Depth (m)	As (µg·L^{-1})	pH	EC (dS·cm^{-1})	CO_3^{2-} (mg·L^{-1})	HCO_3^- (mg·L^{-1})	NO_3^- (mg·L^{-1})	SO_4^{2-} (mg·L^{-1})	Cl^- (mg·L^{-1})	F (mg·L^{-1})
**CW1**	EP	34	126.8	7.53	0.14	36	75.64	954.61	750.83	114.31	ND
**CW2**	EP	34	129.9	7.43	0.17	19.2	73.2	632.54	1028.53	68.87	ND
**CW3**	EP	27	200.7	7.38	0.16	20.4	52.46	1328.42	982.32	276.9	ND
**CW4**	EP	27	108.3	7.12.	0.28	30	68.32	810.83	1447.04	381.27	ND
**CW5**	EP	27	199.8	7.49	0.14	31.2	78.08	1246.56	801.36	163.3	ND
**CW6**	EP	30	73.47	6.81	0.20	21.6	198.86	1336.33	1264.54	215.84	ND
**CW7**	TW	61	23.3	7.31	0.15	25.2	214.72	2229.18	888.62	131.35	ND
**CW8**	TW	64	88.74	7.98	0.18	27.6	75.64	974.55	856.66	203.77	ND
**Vh9**	HP	34	1.97	7.41	0.05	32.4	101.26	934.64	180.49	78.1	ND
**Vh10**	EP	27	4.60	7.48	0.06	8.4	89.06	1573.62	307.31	93.72	ND
**Vh11**	TW	67	53.48	8.06	0.06	14.4	71.98	1204.15	157.98	42.6	ND
**Vh12**	EP	30	12.23	7.42	0.08	25.2	53.68	1463.54	247.64	115.73	ND
**Vh13**	EP	27	6.08	7.52	0.07	20.4	80.52	ND	ND	100.11	ND
**Vh14**	HP	27	7.70	7.31	0.49	12	54.9	1449.96	104.28	73.13	ND
**Vh15**	EP	37	120.9	7.31	0.06	38.4	73.2	1336.32	210.06	136.32	ND
**Vh16**	HP	34	4.35	7.35	0.09	14.4	109.8	2029.89	308.63	136.32	ND
**Vh17**	HP	37	34.94	7.50	0.08	25.2	71.98	1794.68	481.95	94.43	ND
**Vh18**	TW	96	30.61	7.54	0.11	12	52.46	1469.92	180.82	39.76	ND
**Vh19**	HP	37	18.61	7.57	0.09	18	71.98	1938.63	594.98	136.32	ND
**Vh20**	TW	98	134	7.76	0.11	14.4	109.8	2072.55	232.02	41.18	ND
**Vh21**	EP	24	144.3	7.61	0.05	22.8	43.92	1268.68	182.99	61.06	ND
**Vh22**	EP	27	95.28	7.55	0.06	96	39.04	1352.89	241.15	47.57	ND
**Vh23**	EP	27	12.4	7.20	0.12	38.4	104.92	1719.70	134.70	217.97	ND
**Vh24**	EP	34	7.67	7.49	0.08	31.2	146.40	675.39	312.83	107.92	ND
**Vh25**	EP	30	24.88	7.53	0.05	20.4	165.92	37.04	173.32	64.61	ND
**Vh26**	EP	30	12.89	7.51	0.16	108	276.94	1944.19	516.02	133.48	ND
**Vh27**	EP	30	32.63	7.14	0.07	10.8	86.62	2209.61	394.22	217.97	0.09

Table S2. *Cont.*

Sample Name	Sample Source	Depth (m)	As (µg·L^{-1})	pH	EC (dS·cm^{-1})	CO_3^{2-} (mg·L^{-1})	HCO_3^- (mg·L^{-1})	NO_3^- (mg·L^{-1})	SO_4^{2-} (mg·L^{-1})	Cl^- (mg·L^{-1})	F (mg·L^{-1})
**Vh28**	TW	107	80.03	7.73	0.03	12	32.94	656.58	85.75	106.5	ND
**Vh29**	EP	30	1.33	7.52	0.11	15.6	240.34	1300.73	445.54	80.94	ND
**Vh30**	EP	30	52.87	7.65	0.08	21.6	70.76	1610.51	752.45	121.41	ND
**Vh31**	EP	34	46.47	7.60	0.08	36	62.22	1131.09	340.83	76.68	ND
**Vh32**	EP	37	9.92	7.66	0.09	10.8	222.04	1486.55	469.49	95.85	ND
**Vh33**	EP	34	19.36	7.68	0.12	8.4	106.14	1918.11	215.88	9.94	ND
**Vh34**	EP	24	143	7.61	0.06	22.8	36.6	2364.95	143.90	7.1	ND
**Vh35**	EP	27	41.66	7.21	0.15	7.2	32.94	1028.37	171.80	83.78	0.04
**Vh36**	EP	34	5.75	7.43	0.15	14.4	45.14	1097.70	174.35	60.35	ND
**RYK 37**	HP	20	8.78	7.85	0.34	45.6	9.76	971.85	410.11	546.7	ND
**RYK 38**	HP	18	5.82	7.8	0.04	32.4	32.94	5370	92.28	220.1	ND
**RYK 39**	HP	21	2.52	8.35	0.04	24	95.16	ND	141.67	142.71	ND
**RYK 40**	HP	23	3.01	8.0	0.16	20.4	64.66	612.45	400.51	291.1	ND
**RYK 41**	HP	24	25.45	8.07	0.14	18	61	1193.52	680.49	454.4	ND
**RYK 42**	HP	27	3.99	7.3	0.16	32.4	132.98	1156.18	812.76	901.7	ND
**RYK 43**	HP	30	4.47	7.25	0.15	16.8	46.36	1528.41	429.45	497	ND
**RYK 44**	HP	9	2.67	7.1	0.05	24	48.80	4050	148.68	104.37	0.045
**RYK 45**	HP	14	3.01	7.55	0.08	81.6	64.66	1613.98	176.69	211.58	ND
**RYK 46**	HP	12	5.75	7.2	0.08	21.6	98.82	2235.94	152.07	306.72	ND
**RYK 47**	HP	17	11.33	7.8	0.07	24	30.50	1199.82	189.88	96.56	ND
**RYK 48**	HP	18	7.13	8.05	0.14	38.4	50.02	3540	190.79	418.9	ND
**RYK 49**	HP	23	13.13	7.4	0.07	7.2	90.28	3630	210.69	511.2	ND
**RYK 50**	HP	23	8.92	7.5	0.11	15.6	37.82	3750	1081.88	265.54	ND
**RYK 51**	HP	24	4.23	7.85	0.07	55.2	59.78	2365.28	197.85	66.74	ND
**RYK 52**	HP	26	5.32	8.2	0.03	13.2	82.96	2314.71	82.16	60.35	ND
**RYK 53**	HP	24	17.58	8.3	0.09	24	48.80	3480	394.11	545.99	ND
**RYK 54**	HP	26	31.05	7.8	0.02	33.6	50.02	2131.31	101.94	46.15	ND

Table S2. *Cont.*

Sample Name	Sample Source	Depth (m)	As ($\mu g \cdot L^{-1}$)	pH	EC ($dS \cdot cm^{-1}$)	CO_3^{2-} ($mg \cdot L^{-1}$)	HCO_3^- ($mg \cdot L^{-1}$)	NO_3^- ($mg \cdot L^{-1}$)	SO_4^{2-} ($mg \cdot L^{-1}$)	Cl^- ($mg \cdot L^{-1}$)	F ($mg \cdot L^{-1}$)
**RYK 55**	TW	46	27	7.4	0.07	64.8	53.68	2950.52	183.68	170.4	ND
**RYK 56**	HP	24	7.40	7.7	0.03	26.4	28.06	1567.42	99.68	39.05	ND
**RYK 57**	HP	24	9.73	7.3	0.04	32.4	63.44	2881.29	20.34	47.57	ND
**RYK 58**	HP	15	1.66	7.21	0.03	14.4	30.5	1297.52	86.40	61.77	ND
**RYK 59**	HP	18	7.80	6.9	0.05	57.6	36.60	2650.61	80.01	97.27	ND
**RYK 60**	HP	21	2.58	7.3	0.04	31.2	62.22	3330	84.12	68.87	ND
**RYK 61**	HP	23	14.67	7.6	0.03	34.8	51.24	0	107.96	51.12	ND
**RYK 62**	HP	23	4.21	7.8	0.06	85.2	29.28	2682.83	238.69	124.96	ND
**Sample Name**	**Sample Source**	**Depth (m)**	**Fe ($mg \cdot L^{-1}$)**	**Si ($mg \cdot L^{-1}$)**	**P ($mg \cdot L^{-1}$)**	**B ($mg \cdot L^{-1}$)**	**Al ($mg \cdot L^{-1}$)**	**Ca ($mg \cdot L^{-1}$)**	**Mg ($mg \cdot L^{-1}$)**	**Na ($mg \cdot L^{-1}$)**	**K ($mg \cdot L^{-1}$)**
**CW1**	EP	34	0.14	6.4	0.04	0.64	0.001	36.34	19.43	0.24	11.29
**CW2**	EP	34	0.12	7.89	ND	0.27	0.0002	77.08	37.13	363.2	14.11
**CW3**	EP	27	0.17	8.43	0.01	0.13	0.0025	110.9	44.71	252.3	14.33
**CW4**	EP	27	0.56	8.4	0.02	0.27	0.027	88.76	36.82	364.2	14.42
**CW5**	EP	27	0.15	8.11	ND	0.52	0.002	59.89	36.94	332.6	10.97
**CW6**	EP	30	0.19	9.79	0.04	0.65	0.001	65.71	42.1	397.8	17.19
**CW7**	TW	61	0.12	7.19	ND	0.65	0.0003	45.52	24.2	373	35.76
**CW8**	TW	64	0.12	9.38	ND	0.58	0.00008	53.51	45.7	384.8	15.01
**Vh9**	HP	34	0.62	9.12	ND	0.12	0.001	56.48	17.07	73.59	7.06
**Vh10**	EP	27	0.09	9.47	ND	0.12	0.0009	61.2	24.55	65.5	7.81
**Vh11**	TW	67	0.13	9.23	ND	0.12	0.0001	52.92	18.22	70.9	32.09
**Vh12**	EP	30	0.09	7.92	ND	0.19	0.0004	41.92	24.28	131.3	9.06
**Vh13**	EP	27	0.12	8.66	ND	0.13	0.0002	55.66	34.60	80.92	10.53
**Vh14**	HP	27	1.93	5.71	ND	0.04	0.038	39.33	13.19	32.77	4.78
**Vh15**	EP	37	0.14	8.28	ND	0.18	0.0004	59.48	21.96	103.1	9.11
**Vh16**	HP	34	0.21	8.81	ND	0.22	ND	47.57	34.68	155.3	10.86
**Vh17**	HP	37	0.17	8.37	ND	0.24	0.0001	89.97	30.62	139	10.44

Table S2. *Cont.*

Sample Name	Sample Source	Depth (m)	Fe ($mg·L^{-1}$)	Si ($mg·L^{-1}$)	P ($mg·L^{-1}$)	B ($mg·L^{-1}$)	Al ($mg·L^{-1}$)	Ca ($mg·L^{-1}$)	Mg ($mg·L^{-1}$)	Na ($mg·L^{-1}$)	K ($mg·L^{-1}$)
**Vh18**	TW	96	0.16	8.01	ND	0.04	0.0003	29.3	2.96	10.15	1.51
**Vh19**	HP	37	0.23	7.49	0.003	0.24	0.0001	91.98	31.94	162.4	12.2
**Vh20**	TW	98	0.16	7.46	ND	0.18	0.0007	61.57	15.14	105.7	8.04
**Vh21**	EP	24	0.12	8.91	ND	0.13	0.0006	64.43	17.26	77.95	7.40
**Vh22**	EP	27	0.11	8.62	ND	0.12	0.0016	55.29	15.3	75.72	7.48
**Vh23**	EP	27	0.10	9.42	ND	0.24	0.0007	48.37	37.48	198.6	23.21
**Vh24**	EP	34	0.16	7.95	ND	0.19	0.0003	72.38	30.4	110.3	13.86
**Vh25**	EP	30	0.12	9.20	ND	0.13	0.0003	51.55	19.04	73.79	9.31
**Vh26**	EP	30	0.12	10.10	ND	0.17	0.0006	89.11	60.94	160.7	84.4
**Vh27**	EP	30	0.11	7.69	ND	0.07	0.0006	67.31	32.76	109.7	9.07
**Vh28**	TW	107	0.10	9.41	ND	0.10	0.0008	33.72	21	48.36	8.37
**Vh29**	EP	30	0.22	7.80	ND	0.38	0.0008	82.07	41.32	175.7	10.2
**Vh30**	EP	30	0.20	9.16	ND	0.17	0.0003	86	33.92	114.4	8.98
**Vh31**	EP	34	0.14	8.50	ND	0.21	0.0016	59.36	25.98	117.4	7.41
**Vh32**	EP	37	0.11	4.95	ND	0.39	0.0020	35.84	25.09	181.7	8.85
**Vh33**	EP	34	0.12	9.04	0.007	0.26	0.0019	55.16	35.96	202.6	23.22
**Vh34**	EP	24	0.16	7.46	0.003	0.48	0.0038	49.26	22.81	271.5	11.95
**Vh35**	EP	27	1.49	9.37	0.005	0.12	0.037	86.62	34.15	90.96	8.64
**Vh36**	EP	34	0.12	10.38	ND	0.18	0.0007	84.1	62.98	157	85.21
**RYK 37**	HP	20	0.22	10.03	ND	0.85	ND	99.27	71.32	1017	21.76
**RYK 38**	HP	18	0.52	14.91	ND	0.09	ND	26.7	13.96	65.09	6.52
**RYK 39**	HP	21	0.09	9.07	ND	0.11	0.0007	32.96	14.72	41.92	9.07
**RYK 40**	HP	23	0.21	6.39	ND	0.67	0.00006	79.71	66.90	449.6	17.2
**RYK 41**	HP	24	0.30	7.52	0.03	0.50	0.00057	84.25	55.39	571.1	31.25
**RYK 42**	HP	27	0.62	9.81	0.008	1.43	0.00009	228.2	82.24	1242	22.36
**RYK 43**	HP	30	0.24	7.63	ND	0.28	0.00008	178.5	58.69	632.3	28.65
**RYK 44**	HP	9	0.12	7.72	ND	0.14	0.00017	30.59	19.26	103.4	9.41

Table S2. *Cont.*

Sample Name	Sample Source	Depth (m)	Fe ($mg·L^{-1}$)	Si ($mg·L^{-1}$)	P ($mg·L^{-1}$)	B ($mg·L^{-1}$)	Al ($mg·L^{-1}$)	Ca ($mg·L^{-1}$)	Mg ($mg·L^{-1}$)	Na ($mg·L^{-1}$)	K ($mg·L^{-1}$)
**RYK 45**	HP	14	0.23	10.3	ND	0.12	0.00004	85.22	36.13	96.52	22.59
**RYK 46**	HP	12	0.16	8.49	ND	0.18	0.00006	36.03	38.61	156.8	26.8
**RYK 47**	HP	17	0.21	10.78	0.005	0.16	0.00033	54.06	27.22	111.6	19.56
**RYK 48**	HP	18	0.19	6.95	ND	0.22	ND	31.88	46.30	232.7	9.32
**RYK 49**	HP	23	0.21	17.51	0.008	0.21	0.00047	52.57	29.93	137.5	17.17
**RYK 50**	HP	23	0.36	10.77	0.005	0.44	0.00007	140	80.98	206.8	10.57
**RYK 51**	HP	24	0.21	8.79	0.002	0.24	0.00032	18.69	19.83	149.4	4.73
**RYK 52**	HP	26	0.11	9.02	ND	0.12	0.00054	15.66	8.95	63.44	3.98
**RYK 53**	HP	24	0.15	9.91	ND	0.17	0.00013	87.39	37.17	306.3	8.42
**RYK 54**	HP	26	0.27	5.31	0.068	0.19	0.00108	49.1	19.80	114.2	11.39
**RYK 55**	TW	46	0.35	10.55	ND	0.24	0.00007	33.61	25.78	204.9	8.53
**RYK 56**	HP	24	0.19	9.34	ND	0.08	0.00003	31.53	9.60	39.63	3.61
**RYK 57**	HP	24	0.22	5.94	0.01	0.13	0.00043	35.63	22.75	83.54	8.90
**RYK 58**	HP	15	0.15	3.86	ND	0.05	0.00019	48.4	12.65	26.92	4.22
**RYK 59**	HP	18	0.33	7.87	ND	0.09	ND	32.7	31.41	69.42	8.09
**RYK 60**	HP	21	0.10	7.4	ND	0.07	0.00055	29.94	18.96	44.23	5.27
**RYK 61**	HP	23	0.25	8.80	ND	0.07	ND	33.11	14.32	39.76	5.60
**RYK 62**	HP	23	0.15	8.19	ND	0.25	0.00021	14.08	8.84	214.2	8.12

As, Arsenic; EC, electrical conductivity; CO_3^{2-}, Carbonates; HCO_3^-, Bicarbonates; NO_3^-, Nitrate; SO_4^{2-}, Sulfate; Cl^-, Chloride; F, Fluoride; Fe, Iron; Si, Silicon; P, Phosphorus; B, Boron; Al, Aluminum; Ca, Calcium; Mg, Magnesium; Na, Sodium; K, Potassium; CW, Chichawatni; Vh, Vehari; RYK, Rahim Yar Khan; EP, Electric Pump; HP, Hand Pump; TW, Tube Well; ±S.D, ±Standard Deviation; ND, Not Detected.

Table S3. Maximum permissible limits for As and other drinking water quality parameters set by different organizations.

Parameter	Pak-EPA Limits [a]	WHO Limits [b]	US-EPA Limits [c]	Health Canada [d]
As ($mg \cdot L^{-1}$)	0.05	0.01	0.01	0.01
pH	-	6.5–8.5	-	-
Na ($mg \cdot L^{-1}$)	-	200	-	200
Mg ($mg \cdot L^{-1}$)	-	150	-	50
Al ($mg \cdot L^{-1}$)	0.2	0.2	-	-
Ca ($mg \cdot L^{-1}$)	200	100	-	200
Cr ($mg \cdot L^{-1}$)	0.05	0.05	0.01	0.05
K ($mg \cdot L^{-1}$)	-	12	-	-
Mn ($mg \cdot L^{-1}$)	0.5	-	0.05	0.05
Ni ($mg \cdot L^{-1}$)	0.02	0.02	-	-
Cu ($mg \cdot L^{-1}$)	2	2	1.3	1
Zn ($mg \cdot L^{-1}$)	5	-	5	5
Fe ($mg \cdot L^{-1}$)	-	0.3	0.3	0.3
F ($mg \cdot L^{-1}$)	1.5	1.5	4	-
B ($mg \cdot L^{-1}$)	4	2.4		5
Cd ($mg \cdot L^{-1}$)	0.01	0.003	0.005	0.005
Pb ($mg \cdot L^{-1}$)	0.05	0.001	0.015	0.01
CO_3^{2-} ($mg \cdot L^{-1}$)	500	500	-	-
HCO_3^- ($mg \cdot L^{-1}$)	-	-	-	-
Cl^- ($mg \cdot L^{-1}$)	250	250	250	<251
NO_3^- ($mg \cdot L^{-1}$)	50	50	10	45
SO_4^{2-} ($mg \cdot L^{-1}$)	400	500	500	500

A dash (-) indicates that there is no information available regarding possible limits.

[a] As per Pakistan Environmental Protection Agency (Ministry of Environment), Government of Pakistan. 2008.

[b] As per Canadian or BC Health Act Safe Drinking Water Regulation BC Reg 230/92, & Sch 120, 2001. Task force of the Canadian Council or Resource and Environment Ministers Guidelines for Canadian Drinking Water Quality. 1996.

[c] As per the U.S. Environmental Protection Agency Drinking Water Standards. 2008.

[d] As per the WHO guidelines for drinking water quality, 2nd edition. Geneva, World Health Organization. 2008.

Table S4. Concentration of drinking water quality parameters in groundwater samples collected from three rural areas of Punjab, Pakistan.

Sample Name	Sample Source	Depth (m)	Pb ($mg \cdot L^{-1}$)	Cd ($mg \cdot L^{-1}$)	Cu ($mg \cdot L^{-1}$)	Cr ($mg \cdot L^{-1}$)	Co ($mg \cdot L^{-1}$)	Ni ($mg \cdot L^{-1}$)	Zn ($mg \cdot L^{-1}$)	Mn ($mg \cdot L^{-1}$)	S ($mg \cdot L^{-1}$)	SAR
CW1	EP	34	0.004	0.0003	0.025	0.001	0.0003	0.006	0.24	0.11	106.1	0.006
CW2	EP	34	0.0008	0.00002	0.005	0.0003	0.0005	0.004	0.02	0.20	226	6.81
CW3	EP	27	0.003	0.0002	0.020	0.0008	0.0008	0.009	0.18	0.49	173.7	4.04
CW4	EP	27	0.006	0.0001	0.031	0.0012	0.0007	0.006	0.17	0.23	224.9	6.49
CW5	EP	27	0.0014	0.0001	0.008	0.0008	0.0005	0.004	0.06	0.26	184.4	6.81
CW6	EP	30	0.0055	0.0004	0.04	0.0017	0.0005	0.01	0.37	0.20	181.2	7.73
CW7	TW	61	0.0009	0.0001	0.004	0.0006	0.0002	0.002	0.02	0.13	156.8	8.97
CW8	TW	64	0.0015	0.0001	0.009	0.0015	0.0004	0.003	0.03	0.05	159.3	7.84
Vh9	HP	34	0.005	0.00008	0.04	0.0006	0.0007	0.007	0.4	0.22	25.55	1.70
Vh10	EP	27	0.001	0.00003	0.003	0.0006	0.0004	0.003	0.02	0.02	33.82	1.41
Vh11	TW	67	0.002	0.0001	0.07	0.0014	0.0004	0.004	0.06	0.12	25.66	1.67
Vh12	EP	30	0.001	0.00009	0.009	0.0005	0.0003	0.003	0.05	0.23	45.82	3.24
Vh13	EP	27	0.0009	ND	0.003	0.0005	0.0004	0.004	0.02	0.20	31	1.71
Vh14	HP	27	0.015	0.00003	0.21	0.0015	0.0004	0.006	0.53	0.13	10.57	0.90
Vh15	EP	37	0.0017	0.00002	0.007	0.0007	0.0004	0.005	0.04	0.26	36.64	2.27
Vh16	HP	34	0.0041	0.00000	0.11	0.0008	0.0007	0.004	1.89	0.29	51.18	3.46
Vh17	HP	37	0.0005	0.00001	0.003	0.0005	0.0005	0.006	0.01	0.77	95.05	2.52
Vh18	TW	96	0.0005	ND	0.001	ND	0.0001	0.001	0.03	0.03	6.29	0.35
Vh19	HP	37	0.0032	0.0001	0.019	0.0007	0.0006	0.008	0.18	0.63	102.8	2.90
Vh20	TW	98	0.0012	0.00002	0.011	0.0005	0.0004	0.005	0.05	0.53	49.9	2.39
Vh21	EP	24	0.0027	0.00008	0.007	0.0006	0.0004	0.004	0.06	0.26	43.24	1.71
Vh22	EP	27	0.0019	0.00002	0.003	0.0009	0.0004	0.003	0.02	0.11	31.7	1.78
Vh23	EP	27	0.001	0.00001	0.003	0.0006	0.0006	0.003	0.01	0.06	53.27	4.34
Vh24	EP	34	0.0007	ND	0.0019	0.0005	0.0004	0.003	0.02	0.34	62.42	2.17
Vh25	EP	30	0.0026	0.00002	0.009	0.0008	0.0003	0.003	0.06	0.22	29.78	1.75
Vh26	EP	30	0.0015	0.00004	0.024	0.0008	0.002	0.007	0.01	0.16	78.43	2.65
Vh27	EP	30	0.0024	0.00004	0.013	0.0006	0.0004	0.004	0.05	0.05	47.18	2.19

Table S4. *Cont.*

Sample Name	Sample Source	Depth (m)	Pb ($mg \cdot L^{-1}$)	Cd ($mg \cdot L^{-1}$)	Cu ($mg \cdot L^{-1}$)	Cr ($mg \cdot L^{-1}$)	Co ($mg \cdot L^{-1}$)	Ni ($mg \cdot L^{-1}$)	Zn ($mg \cdot L^{-1}$)	Mn ($mg \cdot L^{-1}$)	S ($mg \cdot L^{-1}$)	SAR
Vh28	TW	107	0.0021	0.00004	0.007	0.0007	0.0003	0.002	0.06	0.05	15.73	1.31
Vh29	EP	30	0.01	0.000016	0.005	0.0005	0.001	0.005	0.01	0.41	102.3	3.17
Vh30	EP	30	0.0017	0.00006	0.009	0.0005	0.0005	0.005	0.06	0.32	75.55	2.08
Vh31	EP	34	0.0011	0.000002	0.004	0.0008	0.0004	0.003	0.02	0.26	57.34	2.54
Vh32	EP	37	0.0017	0.0002	0.004	0.0005	0.0004	0.002	0.03	0.20	92.19	4.70
Vh33	EP	34	0.003	0.0001	0.02	0.0009	0.0007	0.007	0.18	0.09	57.73	4.28
Vh34	EP	24	0.004	0.0001	0.01	0.001	0.0005	0.009	0.06	0.05	128.3	6.40
Vh35	EP	27	0.005	0.000003	0.01	0.0009	0.0008	0.005	0.07	0.45	25.87	1.65
Vh36	EP	34	0.002	0.00003	0.02	0.0008	0.0029	0.007	0.01	0.14	84.21	2.62
RYK 37	HP	20	0.0010	0.000003	0.006	0.0005	0.0006	0.006	0.04	0.09	526.7	15.75
RYK 38	HP	18	0.0016	ND	0.008	0.0005	0.0003	0.003	0.27	0.04	16.05	2.05
RYK 39	HP	21	0.0009	0.00004	0.002	0.0003	0.0001	0.001	0.15	0.01	33.39	1.21
RYK 40	HP	23	0.0017	0.00001	0.03	0.0005	0.0004	0.005	0.09	0.05	321.7	7.53
RYK 41	HP	24	0.0068	0.0003	0.03	0.001	0.0009	0.01	0.40	0.20	309.2	9.75
RYK 42	HP	27	0.012	0.0002	0.02	0.0007	0.0014	0.014	0.43	0.19	826.8	14.05
RYK 43	HP	30	0.002	0.00010	0.02	0.0006	0.0011	0.01	0.45	0.25	308.8	8.17
RYK 44	HP	9	0.001	ND	0.004	0.0006	0.0001	0.002	0.10	0.004	31.76	2.95
RYK 45	HP	14	0.002	ND	0.006	0.0009	0.0005	0.005	0.10	0.06	84.38	1.75
RYK 46	HP	12	0.005	0.00008	0.07	0.001	0.0003	0.006	0.23	0.09	65.11	3.70
RYK 47	HP	17	0.004	0.00017	0.028	0.0009	0.0004	0.006	0.32	0.08	70.69	2.48
RYK 48	HP	18	0.001	0.00001	0.005	0.0005	0.0001	0.003	0.14	0.04	63.68	5.41
RYK 49	HP	23	0.007	0.00020	0.049	0.001	0.0005	0.008	0.50	0.11	67.3	3.04
RYK 50	HP	23	0.004	0.00016	0.02	0.001	0.001	0.01	0.43	0.21	273.4	2.80
RYK 51	HP	24	0.015	0.00013	0.17	0.001	0.0001	0.004	0.44	0.01	54.37	4.91
RYK 52	HP	26	0.003	0.00005	0.02	0.0008	ND	0.001	0.38	0.01	18.38	2.57
RYK 53	HP	24	0.0024	0.00006	0.015	0.0007	0.0004	0.006	0.14	0.10	196.7	5.48
RYK 54	HP	26	0.0089	0.00056	0.05	0.001	0.0005	0.014	1.52	0.08	50.18	2.74

Table S4. *Cont.*

Sample Name	Sample Source	Depth (m)	Pb ($mg\cdot L^{-1}$)	Cd ($mg\cdot L^{-1}$)	Cu ($mg\cdot L^{-1}$)	Cr ($mg\cdot L^{-1}$)	Co ($mg\cdot L^{-1}$)	Ni ($mg\cdot L^{-1}$)	Zn ($mg\cdot L^{-1}$)	Mn ($mg\cdot L^{-1}$)	S ($mg\cdot L^{-1}$)	SAR
RYK 55	TW	46	0.0046	0.00002	0.03	0.0008	0.0002	0.004	0.14	0.06	77.19	5.38
RYK 56	HP	24	0.0007	ND	0.001	0.0005	0.0001	0.001	0.07	0.02	15.24	1.22
RYK 57	HP	24	0.005	0.0002	0.025	0.001	0.0003	0.008	0.40	0.03	32.66	2.20
RYK 58	HP	15	0.0025	ND	0.007	0.0003	0.0002	0.005	0.25	0.02	18.6	0.68
RYK 59	HP	18	0.0011	ND	0.003	0.0005	0.0001	0.002	0.14	0.05	25.67	1.76
RYK 60	HP	21	0.0032	ND	0.002	0.0004	0.0001	0.001	0.09	0.040	13.66	1.27
RYK 61	HP	23	0.0017	0.000017	0.005	0.0002	0.0002	0.004	0.23	0.10	19.63	1.15
RYK 62	HP	23	0.0009	ND	0.002	0.0007	0.0001	0.001	0.05	0.01	52.35	9.02

Pb, Lead; Cd, Cadmium; Cu, Copper; Cr, Chromium; Co, Cobalt; Ni, Nickel; Zn, Zinc; Mn, Manganese; S, Sulfur; SAR, Sodium Adsorption Ratio; CW, Chichawatni; Vh, Vehari; RYK, Rahim Yar Khan; EP, Electric Pump; HP, Hand Pump; TW, Tube Well; ±S.D, ±Standard Deviation; ND, Not Detected.

References

1. Rahman, M.M.; Naidu, R.; Bhattacharya, P. Arsenic contamination in groundwater in the Southeast Asia region. *Environ. Geochem. Health* **2009**, *31*, 9–21.
2. Naidu, R.; Smith, E.; Owens, G.; Bhattacharya, P.; Nadebum, P. Arsenic around the world—An overview. In *Managing Arsenic in the Environment: From Soil to Human Health*; CSIRO publishing: Victoria, Australia, 2006; Volume 3–31.

© 2015 by the authors; licensee MDPI, Basel, Switzerland. This article is an open access article distributed under the terms and conditions of the Creative Commons Attribution license (http://creativecommons.org/licenses/by/4.0/).
